# Corticosterone Under Experimental Manipulation of Nutrition and Parasite Burden in a Wild Rodent System

**DOI:** 10.1002/jez.70108

**Published:** 2026-06-23

**Authors:** Sarah E. Wolf, Olga Dłużniewska, Simon A. Babayan, Amy B. Pedersen, Tom J. Little

**Affiliations:** ^1^ Institute of Ecology and Evolution, School of Biological Science University of Edinburgh Edinburgh UK; ^2^ School of Biodiversity, One Health and Veterinary Medicine University of Glasgow Glasgow UK

**Keywords:** anthelmintic, glucocorticoids, macroparasites, nematode, stress, wood mouse

## Abstract

Environmental pressures shape survival and life‐history dynamics partly by triggering the release of glucocorticoids, whose short‐term benefits but long‐term costs make it essential to understand what drives their levels. Here, we used an experimental manipulation of two environmental stressors—food availability and parasite burden—to directly test how they influence fecal corticosterone metabolites (FCMs) in wild wood mice (*Apodemus sylvaticus*). To do so, we experimentally altered nutrition via high‐quality food supplementation and reduced gastrointestinal nematode infection by anthelmintic treatment, using *Heligmosomoides polygyrus* as an indicator of treatment efficacy. FCM levels were not impacted by either treatment. However, our results may be mediated by variation in resource availability or masked by other factors that affect corticosterone. For example, FCMs declined seasonally, alongside a decline in the number of reproducing individuals. While we expected higher food availability and lower worm burdens to decrease stress, putatively higher rates of reproduction in food‐supplemented areas may offset potential declines in corticosterone. Thus, alleviating some environmental stressors in the wild may have unintended consequences on host fitness.

## Highlights

- Food supplementation and anthelmintic treatment did not affect fecal corticosterone metabolites (FCMs) in wild wood mice.
- FCMs declined seasonally and were higher in adults and females.
- Food‐induced reproduction may offset effects of reduced stress.

## 1 Introduction

Environmental challenges shape survival and life‐history traits in wild populations (Defolie et al. 2020), yet the physiological mechanisms linking simultaneous stressors to fitness remain incompletely understood. Glucocorticoids, steroid hormones regulated by the hypothalamic–pituitary–adrenal (HPA) axis, play a central role in mediating these effects, reallocating energy among competing functions such as growth, maintenance, reproduction, and immune defense (Defolie et al. 2020; Patterson et al. 2014; Timmermans et al. 2019; Wingfield and Sapolsky 2003). As such, glucocorticoids provide a key mechanistic link between environmental conditions and life‐history trade‐offs.

In natural systems, animals are exposed to multiple stressors simultaneously, such as nutritional limitation and parasitism, which may influence glucocorticoids in complex and interacting ways. Infection by parasites can trigger the stress response (Defolie et al. 2020; Wensveen et al. 2019), and meta‐analyses show that glucocorticoids often increase following infection (Defolie et al. 2020; O'Dwyer et al. 2020). Similarly, food restriction frequently elevates glucocorticoid concentrations (Heiderstadt et al. 2000; Kitaysky et al. 2001; Levay et al. 2010; Navarro‐Castilla et al. 2019; Romero and Wikelski 2001). Despite these general patterns, empirical findings are inconsistent (Cizauskas et al. 2015; Defolie et al. 2020; Monello et al. 2010; St. Juliana et al. 2014), and it remains unclear whether these stressors act independently, additively, or interactively in shaping endocrine responses in the wild. Experimental evidence suggests potential interactions: for example, in the rodent *Peromyscus* spp., removal of intestinal parasites reduces glucocorticoid levels, with further reductions when food was supplemented (Pedersen and Greives 2008). However, such integrative tests remain rare.

Crucially, responses to environmental stressors are not uniform across individuals. Glucocorticoid secretion varies with season and reproductive state (Michael Romero 2002), and is modulated by sex hormones, with estrogens generally stimulating and androgens suppressing HPA axis activity (Heck and Handa 2019; Kalil et al. 2013). In females, glucocorticoid levels can also fluctuate across the estrogenic cycle (Pilorz et al. 2009) and increase during metabolically taxing reproductive stages (Edwards and Boonstra 2018; Pofi and Tomlinson 2020). Such sex differences may also reflect behavioral and ecological variation, including differences in foraging effort, social interactions, and reproductive investment (e.g., Hernández et al. 2019; Trainor et al. 2010). Glucocorticoid profiles can also shift with age (Heidinger et al. 2008; Wada 2008; Wilcoxen et al. 2011), reflecting changes in reproductive investment and adrenal function. Altogether, failure to account for these intrinsic sources of variation may obscure how environmental stressors influence endocrine responses in the wild.

Here, we address these gaps using a field experiment in wild wood mice (*Apodemus sylvaticus*). We manipulated (i) food availability via supplementation and (ii) parasite burden by reduction of the gastrointestinal nematode *Heligmosomoides polygyrus* with anthelmintic treatment (Gregory et al. 1990; Pedersen and Antonovics 2013). Glucocorticoid levels were assessed via fecal corticosterone metabolites (FCMs), a noninvasive measure that captures circulating levels over hours to days (Kalliokoski et al. 2010; Karaer et al. 2023; Palme 2019). We predicted that (1) food supplementation and parasite removal would each reduce FCMs, and/or (2) their combined effects could be additive. To better isolate these treatment effects—and to quantify general patterns of glucocorticoid secretion—we incorporated factors including reproductive status, age, and sex. Because the study was conducted during the breeding season, individuals may have redirected any energetic or nutritional benefits gained from our treatments towards reproductive investment (e.g., mating effort or offspring production) rather than improving body condition, potentially obscuring any expected decrease in FCM concentrations.

## 2 Materials and Methods

### 2.1 Ethics Statement

All animal work was carried out under the approved UK Home Office Project License PP0798440 in accordance with the UK Home Office, in compliance with the Animals (Scientific Procedures) Act 1986 and approved by the University of Edinburgh Animal Welfare and Ethical Review Body (AWERB). Fieldwork was performed with permission from landowners.

### 2.2 Field Work, Mouse Capture, and Sample Collection

Wild wood mice were captured from May 30, 2023, to November 23, 2023, at two forested sites near Edinburgh, Scotland, UK: Penicuik Estates (55.8261°N, 3.2467°W) and Bilston Wood (55.8714°N, 3.1470°W). This period covers the breeding season, with reproductively active individuals most abundant from June to August (see Figure S1). At each site, we established two 80 m × 80 m grids separated by ≥ 50 m; at this distance, no mice were trapped on multiple grids. Each grid contained 64 trap stations (10 m × 10 m), with two Sherman live traps (H.B. Sherman 5.1 × 6.4 × 16.5 cm folding traps, Tallahassee, FL, USA) placed under trap covers to protect animals from inclement weather, for a total of 256 traps set each night.

We live‐trapped each site every 3 weeks for three consecutive nights for a total of 9 trapping sessions (defined as 3 consecutive trapping nights in a week) and 27 total trapping nights. Autoclaved Sherman live traps were set late each afternoon and collected the following morning. Each trap contained bedding and a bait consisting of a mix of seeds, mealworms, and a carrot slice. At first capture, individuals above 12 g were given subcutaneous RFID tags (125 kHz, Francis Scientific Instruments, UK). Individuals smaller than 12 g were given a unique ear biopsy, and when recaptured and were > 12 g, they received an RFID tag.

Across both sites, 317 unique mice were captured. Individuals caught in two or more trapping sessions were classified as residents, resulting in 100 resident mice. Between‐session recapture rate was 45.1%, increasing to 57.7% when considering within‐session recaptures (mouse caught on consecutive days). Analyses of population demographics and fecal egg counts used all 317 mice, whereas FCM analyses focused on residents.

During the first capture per session, several individual traits were recorded before mice were released at their site of capture. Weight (g) and flattened body length (from nose to the base of the tail, mm) were measured. Sex was determined based on the appearance of genitalia, with a greater urogenital distance in males. Males with descended or scrotal testes were classified as reproductively active, while those with abdominal (non‐visible) testes were considered nonreproductive, as testis position reflects spermatogenesis in rodents (Herbreteau et al. 2011; Massoud et al. 2021). Females were considered reproductive if pregnant (abdominal swelling) or lactating (enlarged nipples); those with a perforate or non‐perforate vagina were classed as nonreproductive. Note that this method likely misclassifies females in early pregnancy (Herbreteau et al. 2011). Last, age class (juvenile, subadult, and adult) was determined based on body mass (juvenile: < 10 g; subadult: 10–15 g; and adult > 15 g) and fur color, which transitions from gray to brown pelage into adulthood (Herbreteau et al. 2011).

Fecal samples were collected from the Sherman traps. Fecal pellets were weighed and stored in formalin at 4°C until fecal egg counts were performed. When available, an additional 5–10 dry pellets were collected in Eppendorf tubes and frozen at −80°C until fecal steroid extraction. Approximately 98% of samples were collected by 6 p.m. on the day of capture, that is, within ≤ 12 h, with exceptions on heavy trapping days, when a limited number were stored overnight at 4°C and collected the next morning (≤ 48 h). While some microbial modification of FCMs can occur after defecation, the collection timing in this study should limit substantial degradation prior to processing.

### 2.3 Food Supplementation and Drug Treatment

We used a two‐way factorial experimental design to manipulate both food availability and GI nematode burdens. At each of our two sites, one grid was food‐supplemented, and one was given no additional food, resulting in two food‐supplemented and two control grids across the study. On our two food supplemented grids, we distributed 10 kg of TransBreed or Safe 199 high‐quality nutrient veterinary feeds (protein 18.5%, fat 9.5%, starch 36.8%, and high micronutrients) evenly over the grid once a week, starting 2 weeks before the first trapping session. Previous work in this system shows that mice on supplemented grids can exhibit improved body condition, enhanced immune responses, reduced parasite burdens, and shifts in parasite community composition (Erazo et al. 2025; Sweeny et al. 2021). Thus, although individual food intake cannot be quantified, this evidence suggests that supplementation influences physiology.

Parasite treatment was conducted at the individual level. Captured wood mice were randomly assigned at first capture to receive either anthelmintic drug treatment or a water control. We balanced anthelmintic administration across sex and date captured within each of the four grids. Individuals assigned to drug treatment received a combination of 100 mg/kg Pyrantel and 9.4 mg/kg Ivermectin using a sterilized oral gavage. Control individuals received an equal body‐size‐adjusted amount of tap water. Ivermectin and Pyrantel are commonly used anthelmintic drugs that reduce gastrointestinal nematodes (Pedersen and Fenton 2015); this combination of drug treatments reduces *H. polygyrus* burdens by > 99% in wild wood mice for ~16 days (Clerc et al. 2019; Sweeny et al. 2021). Due to the delayed delivery of Pyrantel, drug treatment only included the single drug dose of 9.4 kg/mg Ivermectin during the first trapping session at each site (8.7% of total doses across the season), but after this point, all drug‐treated individuals received the combination of drugs. This dose of Ivermectin alone can reduce the probability of nematode infection by > 70% (Knowles et al. 2012); therefore, we are confident that this small group of individuals experienced significant decreases in *H. polygyrus* after this first dose. Only individuals larger than 12 g received treatment, and both anthelmintic and control treatments were repeatedly given at all subsequent recaptures, with a minimum time between doses of 21 days. Mice received one to seven doses over the field season, depending on recapture rate (mean = 2.42 doses, standard error = 0.24, see Figure S2, S3, Table S1). This design results in four treatment groups: mice captured on control or food‐supplemented grids who are either given water or anthelmintic treatments every 21+ days.

### 2.4 Fecal Egg Counts

Fecal samples were analyzed using a modified cuvette method (adapted from Hayward et al. 2019). First, the samples were homogenized and then spun down (1500 rpm for 2 min) in thin‐walled polypropylene tubes, from which the supernatant was aspirated. The remaining compact fecal matter was thoroughly mixed with a saturated salt solution and spun down again (1500 rpm for 2 min). The thin‐walled tubes were clamped just below the meniscus using medical forceps, and this top layer of liquid containing GI parasite eggs was collected in a small beaker. We allowed oocysts to float onto a coverslip placed on top of the beaker for 20 min, at which time it was placed onto a slide for analysis. *H. polygyrus e*ggs were counted at 10× magnification. Egg counts were adjusted by the weight of the fecal sample to generate an “eggs per gram” of feces (EPGs). EPGs were quantified for all available fecal samples from all animals/captures (*n* = 888, *n* = 1–20 samples per individual).

### 2.5 Fecal Steroid Extraction

Noninvasive analysis of FCMs is now a standard tool for assessing adrenocortical activity (e.g., Monello et al. 2010; Navarro‐Castilla, Barja, et al. 2014; Navarro‐Castilla, Mata, et al. 2014; Palme 2019; Touma et al. 2004). FCMs provide an integrated estimate of circulating corticosterone secretion over preceding hours rather than a single instantaneous value. After a stressor, glucocorticoids are metabolized by the liver and excreted into feces following a species‐specific delay that reflects gut transit time; in rodents, FCMs typically appear several hours after changes in plasma concentrations, with peak excretion often around ~8–15 h post‐stress and detectable levels over the following day in some cases (Kalliokoski et al. 2010; Rowland and Toth 2019).

FCMs were extracted from fecal samples of all resident mice, that is, those captured during two or more trapping sessions. This resulted in 268 total samples from 100 individuals, with 1–8 samples from each individual over the study period. The fecal steroid extraction protocol was adapted from Nomoto and Kansaku (2023) and Veitch et al. (2021). Frozen fecal samples were thawed and dried in a heating block at 70°C and powdered using a pestle homogenizer. Using a Mettler AJ50 analytical scale (precision: 0.0001 g, minimum mass: 0.005 g), we measured 0.005 g of fecal powder per sample (average: 0.005244 ± 0.000166 g, range: 0.0050–0.0058 g), from which the hormone was extracted using 1 mL of freshly prepared 80% (v/v) methanol solution. Fully submerged samples were vortexed for 10 s and shaken overnight at room temperature on a tube shaker (average extraction duration = 15 h 3 min 56 s ± 1 min 30 s). Samples were then centrifuged for 10 min at 10,000 × *g*. A total of 0.8 mL of supernatant was transferred to a new tube, and the liquid was evaporated in a heating block at 70°C to isolate steroids. Extractions were stored at −20°C until the assay, when they were reconstituted in immunoassay buffer (Abcam).

### 2.6 Corticosterone ELISA

We quantified corticosterone and its metabolites in fecal samples using a competitive enzyme‐linked immunosorbent assay (ELISA; Abcam, AB108821, minimum detection: 0.28 ng/mL). Although this kit is primarily designed to measure unmetabolized corticosterone in circulation, we found that it can also be used to assess FCMs. First, only a small proportion of unmetabolized corticosterone is excreted in feces (Touma et al. 2003). Instead, feces are dominated by numerous corticosterone metabolites (e.g., > 20; Touma et al. 2003). The high values observed in our samples are therefore unlikely to originate from unmetabolized corticosterone. Second, in a subset of samples (*n* = 22), corticosterone concentrations measured with the Abcam kit were strongly correlated with those obtained using a dedicated FCM ELISA (Arbor Assays; product #K014; Spearman's *ρ* = 0.80, *p* < 0.0001, see Figures S4 and S5). Abcam values were higher, likely due to differences in antibody cross‐reactivity with corticosterone metabolites. Parallelism between serially diluted fecal extracts and the assay standard curve confirmed the Abcam assay's validity for our samples (see Figure S6 for details).

Following the manufacturer's instructions, absorbance was read at 450 nm with the wavelength correction at 570 nm using FLUOstar Omega microplate reader (BMG Labtech). Corticosterone concentrations were extrapolated from the standard curve generated with a 4‐Parameter fit using Omega Data Analysis software (version 3.32 R5). Corticosterone concentrations were standardized against the weight of fecal samples and presented as ng of fecal corticosterone per g of feces (ng/g). Samples with a high coefficient of variation (CV > 20%) between duplicates were rerun. In total, 7.6% of samples (*n* = 22/290) were excluded due to high CV (despite repeated reruns) or, in a few cases, because concentrations fell below the lower limit of the standard curve.

The average intra‐plate variation was 8.9%, while a pooled sample run across all plates indicated an inter‐plate variation of 39.8%. However, we are confident that our results are not driven by this inter‐plate variation. First, fecal samples were assayed in duplicate, with all samples from the same individual tested on the same plate, unless singular samples had to be remeasured. All plates, except for plates containing sample reruns, were also balanced in terms of food treatment, drug treatment, sex, age, body mass, and date. Second, we partially accounted for inter‐plate variation in all models by adding plate ID as a random effect. Third, we reran all models using plate‐adjusted corticosterone values, in which each sample value was divided by the corticosterone value of its inter‐plate control sample and then multiplied by the plate‐wide average corticosterone value. This analysis did not significantly change model results (see Tables S2, S4, and S6).

### 2.7 Statistical Analyses

Statistical analyses were done in R Statistical Software (R version 4.5.1; R Core Team 2019) using the “lme4” package for linear and general linear mixed‐effects models (Bates et al. 2014). When appropriate, models were checked for normality and homoscedasticity.

We first assessed the effects of food supplementation and anthelmintic treatment on several metrics related to abundance, reproduction, and body condition. First, abundance of *Apodemus* was measured as the minimum number known alive (MNKA) for each of the four treatment combinations (supplementation × anthelmintic treatment) during the trapping sessions denoted as integers one to nine (e.g., May–November 2023, following Wolff 1996). To test for the effects of food supplementation and anthelmintic treatment on *Apodemus* abundance, we used a general linear model assuming a Poisson distribution with a default “log” link function to predict MNKA per treatment combination and trapping session, with fixed effects of food and anthelmintic treatment, and their interaction, as well as trapping session. Second, we tested effects on reproductive status. We used a binomial general linear mixed‐effects model with the default “logit” link, with reproductive status as the response variable and fixed effects of food and anthelmintic treatments, as well as their interaction, sampling date, sex, age, and trapping location, with ID as a random effect. Finally, we tested for effects on two proxies of condition: residual body index (i.e., the difference between an individual's actual and predicted mass: length ratio) and skeletal muscle index (i.e., an estimate of muscle mass), which adjusts an individual's body mass by their body length divided by the population's arithmetic mean body length, and is then scaled by the slope of the standardized major axis regression between body length and weight across the population. For each variable, we ran a linear mixed effects model with fixed effects of food and worm egg burden, as well as their interaction, anthelmintic treatment (to account for other potential side effects of the drug), trapping date, sex, age, reproductive status, and trapping location, with ID as a random effect.

Next, to test the efficacy of our anthelmintic treatment, *H. polygyrus* egg burden (eggs/gram feces) was log(*x* + 1)‐transformed and rounded to the nearest integer. Using this egg burden as count data, we ran a general linear‐mixed effect model assuming a Poisson distribution with the default “log” link. We included food supplementation (no supplementation, supplementation) and anthelmintic treatment (control, anthelmintic) as our main fixed effects, as well as their interaction. Other fixed effects included sampling date, reproductive status (reproductive, nonreproductive), sex (male, female), and age category (non‐adult, adult). Both juveniles and subadults were considered non‐adults. Sampling date was formatted as Julian date, that is, days since January 1, and was scaled from 0 to 1 during modeling to reach model convergence. Additionally, to account for variation between two spatial replicates, we included site as a fixed effect. A random effect of mouse ID accounts for repeated samples from the same individual.

To address how manipulations of food availability and parasite burdens influence levels of corticosterone, we tested for the effect of anthelmintic treatment and food supplementation on FCM levels (ng/g, log‐transformed to achieve a normal distribution). We ran a linear mixed effects model assuming a Gaussian distribution that included fixed effects of food supplementation, worm egg burden, and their interaction, as well as anthelmintic treatment, sampling date, reproductive status, sex, age category, and site. Random effects of mouse ID and ELISA plate ID accounted for repeated samples from the same individual and inter‐plate variation, respectively. Test statistics were calculated using Type II sum of squares and reported with Kenward‐Roger degrees of freedom. Similar linear mixed effects models were run to explore further variation in anthelmintic drug treatment: (1) a model which included only fecal samples collected post‐treatment (i.e., no first captures, as the drug treatment was given on the first capture and therefore its effects on egg burdens would not be detectable until subsequent captures) and one model that replaced anthelmintic treatment with the number of doses received up to the sampling date, to test for a cumulative effect of multiple drug doses (see Tables S3–S6).

## 3 Results

**Figure 1:**
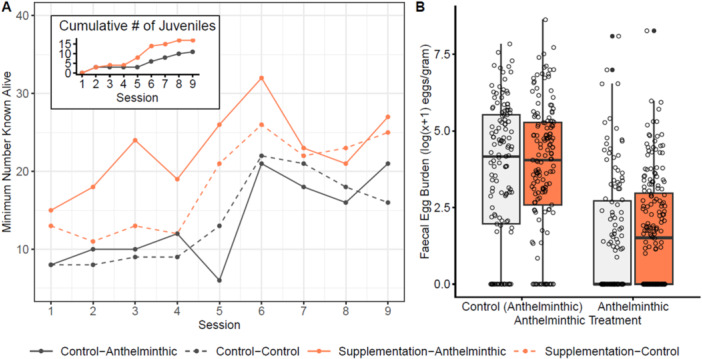
Main treatment effects of food supplementation and anthelmintic treatment. (A) Wood mouse abundance (minimum number known alive) was significantly higher on food‐supplemented grids (orange), regardless of anthelmintic treatment, across trapping sessions (May–November). The inset shows the cumulative number of juveniles found on control (black; *n* = 2 grids) and food‐supplemented (orange; *n* = 2 grids) grids during this period. (B) *Heligmosomoides polygyrus* egg burden (log(*x* + 1)‐transformed eggs per gram of feces) was significantly reduced by anthelmintic treatment.

From May to November 2023, we captured 317 unique wood mice across four grids, of which 286 were large enough to receive treatment with either water or anthelmintic (Ant) drugs (Group sizes: Food‐Ant = 84; Food‐Con = 83; Con‐Ant = 59; and Con‐Con = 60). Food‐supplemented grids had a higher abundance of mice than non‐supplemented grids (*Χ* ^2^ = 25.50, *p* ≤ 0.0001, Figure 1A). Although more juveniles were captured on supplemented grids, this difference was not statistically significant (*Χ* ^2^ = 18.00, *p* = 0.051, Figure 1A). In addition, neither food supplementation nor anthelmintic treatment was associated with the number of reproductive individuals on our grids (supplementation: *Χ* ^2^ = 0.47, *p* = 0.49; anthelmintic: *Χ* ^2^ = 0.06, *p* = 0.80). However, we found that wood mice in reproductive condition were significantly more likely to be found earlier in the season (*Χ* ^2^ = 66.69, *p* < 0.0001) and were more likely to be male (*Χ* ^2^ = 49.14, *p* < 0.0001) and adult (*Χ* ^2^ = 36.55, *p* < 0.0001). In addition, our treatments did not have an effect on residual body index or skeletal muscle index, although female, reproductive, adult individuals had higher body condition‐related indices (Table 1).

**Table 1:** Linear mixed‐effects model of the effect of food supplementation and anthelmintic treatment on wood mouse residual index and skeletal muscle index.

Fixed effects	Residual index				Skeletal muscle index			
	Estimate ± SE	df	*F*	*p*	Estimate ± SE	df	*F*	*p*
Intercept	−1.67 ± 0.85				16.65 ± 0.96			
Food availability	0.06 ± 0.38	1, 332.29	0.05	0.82	−0.16 ± 0.43	1, 326.92	0.29	0.59
Egg burden	0.17 ± 0.08	1, 568.94	7.05	0.008*	0.13 ± 0.09	1, 569.60	4.64	0.03*
Drug treatment	0.38 ± 0.27	1, 248.69	1.92	0.17	0.36 ± 0.31	1, 246.17	1.38	0.24
Julian date^a^	−1.07 ± 0.86	1, 553.79	1.55	0.21	0.79 ± 0.98	1, 551.83	0.66	0.42
Reproductive status	1.78 ± 0.30	1, 569.27	35.78	< 0.0001*	1.12 ± 0.34	1, 568.12	11.03	0.001*
Sex	1.20 ± 0.28	1, 264.05	17.64	< 0.0001*	1.42 ± 0.32	1, 260.73	19.32	< 0.0001*
Age	1.17 ± 0.31	1, 555.93	13.85	0.0002*	0.49 ± 0.35	1, 553.53	1.91	0.17
Site	−0.61 ± 0.27	1, 240.71	4.97	0.03*	−0.58 ± 0.31	1, 237.64	3.47	0.06
Food × Worm burden	−0.46 ± 0.10	1, 568.77	0.19	0.66	0.0001 ± 0.12	1, 569.47	0.00	0.99

*Note:* Data are based on 580 samples from 291 individuals, and mouse ID was included as a random effect. Test statistics are calculated using Type II sum of squares and reported with Kenward–Roger degrees of freedom. Reference levels: no supplementation, no anthelmintic treatment, and nonreproductive, male, and non‐adult individuals.

* *p* < 0.05.

^a^ Julian date is scaled from 0 (end of May) to 1 (end of November).

We assessed 888 fecal samples for *H. polygyrus* egg burden, which ranged from 0 to 5578 eggs per gram of feces (EPG) (mean = 123.69 ± 12.35 [SE] EPG; median = 13.33 EPG). Across all captures during the field season, anthelmintic treatment significantly reduced average *H. polygyrus* egg burden (Table 2, Figure 1B), which was not impacted by food supplementation, sampling date, reproduction, sex, or age. If we compare egg burden between first (baseline; when drug treatment will not yet be effective) and subsequent captures, anthelmintic‐treated mice decreased in average egg burden by 47%, whereas controls increased in average egg burden by 88%. Similarly, anthelmintic‐treated mice exhibited a 17% decrease in *H. polygyrus* prevalence (first capture = 67%, subsequent captures = 55%), whereas control prevalence increased by 25% (first capture = 66%, subsequent captures = 82%), altogether supporting that anthelmintics decreased egg burdens and infection prevalence.

**Table 2:** The effect of food supplementation and anthelmintic treatment on *H. polygyrus* egg burdens in wood mice.

Fixed effects	Estimate ± SE	*z*	*p*
Intercept	0.21 ± 0.39	0.54	0.59
Food availability	0.13 ± 0.16	0.79	0.43
Drug treatment	−0.41 ± 0.18	−2.21	0.03*
Julian date^a^	0.60 ± 0.39	1.53	0.13
Reproductive status	0.03 ± 0.11	2.78	0.78
Sex	0.008 ± 0.13	0.06	0.95
Age	0.14 ± 0.15	0.94	0.35
Site	0.32 ± 0.12	2.53	0.01*
Food × Drug treatment	−0.14 ± 0.23	−0.63	0.53

*Note:* Eggs per gram (EPG) is log(*x* + 1)‐transformed and rounded to the nearest integer before being run in a general linear mixed model with a Poisson distribution. Mouse ID is included as a random effect. Reference levels include: no food supplementation, no anthelmintic treatment, and nonreproductive, male, and non‐adult individuals.

* *p* < 0.05.

^a^ Julian date is scaled from 0 (end of May) to 1 (end of November).

**Figure 2:**
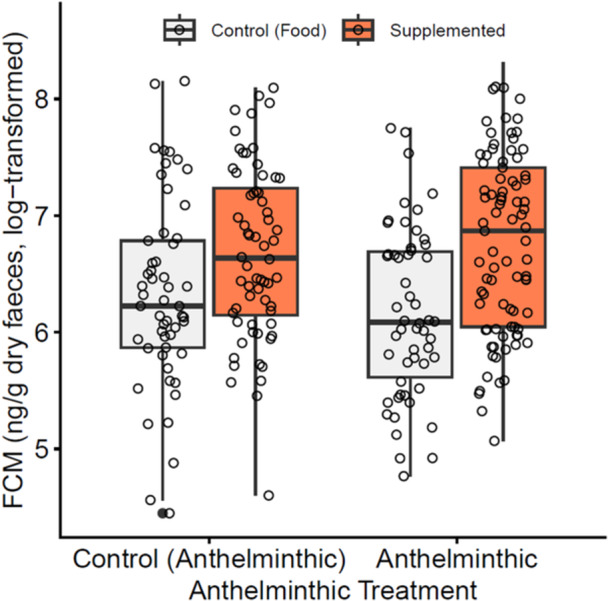
FCM levels following experimental manipulation of food availability (high‐quality mouse chow vs no supplementation) and anthelmintic treatment of *Heligmosomoides polygyrus* (treatment with Ivermectin and Pyrantel vs. water control). The plot is based on data from 268 samples and 100 individuals, and points represent individual samples. FCM concentration is plotted as predicted values from the linear mixed‐effects model based on the log‐transformed concentration (ng/g), and as such, the *y*‐axis begins above 0.

**Figure 3:**
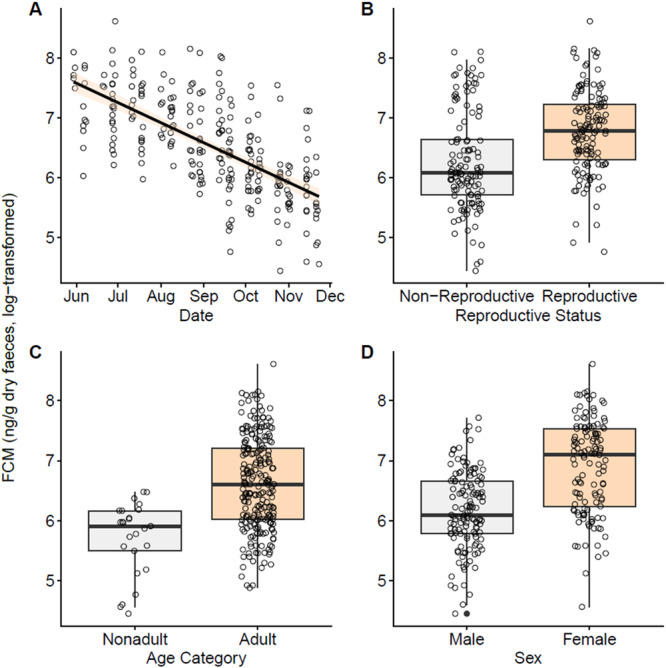
Factors predicting fecal corticosterone metabolites (FCMs). FCM levels were significantly higher earlier in the breeding season (A), marginally in reproductive individuals (B; *p* = 0.051), and in adult (C) and female mice (D). Plots are based on data from 268 samples and 100 individuals, and points represent individual samples. FCM is plotted as predicted values from the linear mixed‐effects model based on the log‐transformed concentration (ng/g); as such, all *y*‐axes start above 0.

We successfully assessed 268 samples for FCMs from 100 resident mice (52 male, 48 female). Fecal corticosterone levels ranged from 35.57 to 23,406.06 ng/g (mean = 1724.98 ± 190.89 ng/g; median = 616.91 ng/g). While FCM levels were somewhat higher in wood mice on food‐supplemented grids (*p* = 0.086, mean_control_ = 6.26, mean_food_ = 6.71 ng/g), we found no evidence for a significant effect of either food supplementation or anthelmintic treatment, nor their interaction (Table 3, Figure 2). Additionally, no effect of anthelmintic treatment was found when only fecal samples collected post‐drug treatment (i.e., only including post‐treatment captures) or when controlling for the number of doses received up to the sampling date (see Tables S3–S6). However, the date of sampling significantly predicted FCMs (Figure 3A), which decreased from June to November. Reproductive mice had higher FCMs than nonreproductive mice; however, this difference was not significant (*p* = 0.052; Figure 3B). We also found that FCM levels were higher in female and adult mice (Figure 3C,D). Finally, there was a spatial effect of the woodland site.

**Table 3:** Linear mixed‐effects model of the effect of food supplementation and anthelmintic treatment on FCMs (log‐transformed ng/g) in wood mice.

Fixed effects	Estimate ± SE	df	*F*	*p*
Intercept	7.71 ± 0.56			
Food availability	−0.01 ± 0.23	1, 100.20	2.99	0.086
*H. polygyrus* egg burden	−0.09 ± 0.05	1, 236.65	1.38	0.24
Drug treatment	−0.10 ± 0.16	1, 75.00	0.37	0.54
Julian date^a^	−2.94 ± 0.53	1, 212.31	29.74	< 0.0001*
Reproductive status	0.33 ± 0.17	1, 213.37	3.79	0.052
Sex	0.58 ± 0.16	1, 83.40	12.08	0.001*
Age	0.73 ± 0.25	1, 210.97	8.37	0.004*
Site	0.32 ± 0.16	1, 72.27	4.05	0.04*
Food × Egg burden	0.10 ± 0.06	1, 233.45	2.57	0.11

*Note:* Data are based on 268 samples from 100 individuals, and mouse ID and assay plate ID were included as random effects. Test statistics are calculated using Type II sum of squares and reported with Kenward–Roger degrees of freedom. Reference levels include: no food supplementation, no anthelmintic treatment, and nonreproductive, male, and non‐adult individuals.

* *p* < 0.05.

^a^ Julian date is scaled from 0 (end of May) to 1 (end of November).

## 4 Discussion

Identifying the drivers of glucocorticoid levels may improve our understanding of variation in survival and fitness. Glucocorticoids integrate multiple processes, including energy demand, immune activity, and responses to environmental challenges. Resource availability and parasite burden may influence FCM levels through several of these non‐mutually exclusive pathways. However, we found no effects of food supplementation, anthelmintic treatment, or their interaction on FCM levels in wild wood mice, contrasting results from a similar manipulation in *Peromyscus* spp. (Pedersen and Greives 2008). One possibility is that natural food availability and parasite burdens during the study period were not sufficiently limiting or physiologically demanding to alter FCM output. Consistent with this interpretation, food supplementation did not substantially improve body condition, suggesting that mice may already have been meeting energetic requirements. Alternatively, our results may be modulated by year‐to‐year variation in resource availability (e.g., tree masting cycles), which we cannot address here, or masked by intrinsic drivers of glucocorticoid secretion, including sex, age, and reproductive activity. Below, we examine the potential for environmental and physiological factors to jointly modulate glucocorticoid levels.

Food supplementation had no detectable effect on FCM levels or body condition, suggesting that energetic limitation in control grids may have been weak or absent. Nevertheless, supplementation may have influenced FCMs indirectly through changes in reproductive activity. FCM levels declined in parallel with a seasonal reduction in reproductive individuals in September (Díaz and Alonso 2003; Moreno and Kufner 1988), and slightly higher FCMs on supplemented grids may reflect increased reproductive effort, immigration, or density‐dependent effects such as competition and parasite exposure (Rogovin et al. (2003); Rogovin et al. 2008). Because supplementation began 2 weeks prior to trapping, we cannot fully exclude pre‐existing differences in density among grids; however, treatments were alternated across grids among years (for another study), and where capture rates differed markedly among years, control grids consistently supported fewer mice. The lack of a significant association between reproductive status and FCMs in our models may also reflect overlapping influences of age and sex, as well as limited detection of early‐stage pregnancies. Glucocorticoids integrate multiple physiological processes, so variation in FCMs likely reflects several interacting mechanisms rather than a single “stress” response.

We also predicted that parasite removal would reduce FCMs, but this was not observed. This aligns with several studies reporting no glucocorticoid response to parasite removal (Carlsson et al. 2016; Monello et al. 2010; Trevisan et al. 2017). One possibility is that observed parasite burdens were relatively well tolerated, or that treatment‐induced reductions were insufficient to affect FCM outputs. Alternatively, removing dominant parasites might alter co‐infection dynamics, potentially increasing other parasites or pathogens (Pedersen and Antonovics 2013) and offsetting any potential reduction in glucocorticoids. Interpretation is further complicated if reproduction tracks food availability, as reproductive individuals often carry higher parasite loads (Shaner et al. 2018), which could confound infection‐stress relationships. Under this scenario, an interaction between anthelmintic treatment and food supplementation might be expected ‐ specifically, reduced FCMs on control grids ‐ but this pattern was not detected.

Males exhibited lower FCM levels than females, consistent with sex differences in other rodent species (Navarro‐Castilla, Barja, et al. 2014; Navarro‐Castilla, Mata, et al. 2014; Navarro‐Castilla et al. 2017, 2019). This pattern likely reflects, in part, sex‐specific modulation of the HPA axis by gonadal steroids, with estrogens stimulating and testosterone suppressing glucocorticoid secretion (Heck and Handa 2019). In females, glucocorticoid levels may also vary with the estrogenic cycle (Atkinson and Waddell 1997; Cavigelli et al. 2005) and increase during energetically costly stages such as pregnancy and especially lactation (Karaer et al. 2023; Palme 2019). More broadly, sex differences may be further shaped by variation in behavior, including foraging effort and social interactions (Hernández et al. 2019; Trainor et al. 2010). As we did not directly measure food intake, estrogenic state, or reproductive status beyond broad categories, we cannot disentangle these mechanisms here; however, our results are consistent with the expectation that females experience greater variation and, on average, higher energetic and physiological demands during the breeding season.

FCM levels also varied among sites, indicating the potential for ecological differences in site quality. Notably, mice at Penicuik exhibited higher FCMs and nematode burdens alongside lower body condition, suggesting that individuals experience poorer overall condition at this site. Such patterns could arise from variation in local resource availability, habitat quality, density, and conditions influencing parasite transmission (Busch and Hayward 2009; Mirante et al. 2025). However, as this study was not designed to identify the drivers of site‐level variation and did not include detailed environmental measurements, these explanations remain speculative. While each site contained all treatment combinations and the site was statistically controlled for, residual ecological heterogeneity among sites may nevertheless have influenced observed patterns in FCM levels.

Clearly, many of the explanations—food availability, seasonality, sex, and even parasitism—for patterns of FCMs lead back to their potential effects on reproduction. In our study, food‐supplemented grids had more mice, which may suggest that reproductive activity was higher, though this may also reflect increased immigration. More accurate molecular assays of pregnancy, accompanied by data on baseline resource availability, would enable progress towards understanding the effects of investment in reproduction on glucocorticoids. Additional measurements could include looking directly at the relationship between FCMs and parasite type, as well as evaluating sex hormones and immune parameters as potential downstream effectors of glucocorticoids.

## Author Contributions

**Sarah E. Wolf:** conceptualization, data curation, formal analysis, investigation, methodology, project administration, supervision, visualization, writing – original draft, writing – review and editing. **Olga Dłużniewska:** data curation, formal analysis, investigation, methodology, visualization, writing – original draft, writing – review and editing. **Simon A. Babayan:** conceptualization, funding acquisition, supervision, writing – review and editing. **Amy B. Pedersen:** conceptualization, funding acquisition, resources, supervision, writing – review and editing. **Tom J. Little:** conceptualization, funding acquisition, investigation, resources, supervision, writing – review and editing.

## Conflicts of Interest

The authors declare no conflicts of interest.

## Supporting information

Supporting File

## Data Availability

The data that support the findings of this study are openly available in Zenodo at https://zenodo.org/, reference number https://doi.org/10.5281/zenodo.19821268 (Wolf et al. 2026).
