# Supplementary material for: Corticosterone Under Experimental Manipulation of Nutrition and Parasite Burden in a Wild Rodent System
Source: J Exp Zool A Ecol Integr Physiol. 2026 Jun 23;345(7):749–58. doi: 10.1002/jez.70108 (PMC13353635; doi:10.1002/jez.70108)
Supplement: Supplementary file 1 — Supporting File [file JEZ-345-749-s001.docx]

Supplemental Materials for **Corticosterone under experimental manipulation of nutrition and parasite burden in a wild rodent system**

| **Table S1. Number of trapping sessions each mouse received treatment (Anthelminthic or Control) over the course of the study. Treatments were given every three or more weeks.** | | | | | | | | | |
| --- | --- | --- | --- | --- | --- | --- | --- | --- | --- |
| **Food** | **Anthelminthic** | **1** | **2** | **3** | **4** | **5** | **6** | **8** | **9** |
| Control | Control | 4 | 10 | 3 | 4 | 0 | 0 | 0 | 0 |
| Control | Anthelminthic | 3 | 11 | 4 | 1 | 1 | 0 | 1 | 0 |
| Food | Control | 5 | 9 | 9 | 2 | 2 | 1 | 0 | 0 |
| Food | Anthelminthic | 4 | 11 | 8 | 4 | 2 | 0 | 0 | 1 |

**Tables S2 – S6:** Linear mixed-effects models of the effect of food supplementation, anthelminthic treatment, and other factors on faecal corticosterone (log-transformed ng/g) in wood mice. For all models, mouse ID and assay plate ID were included as random effects. Test statistics are calculated using Type II sum of squares and reported with Kenward-Roger degrees of freedom. Reference levels: no food supplementation, no anthelminthic treatment, and non-reproductive, male, and non-adult individuals.

Models vary in two main ways:

1. Tables S3 – S6 aim to further explore the predictive power of anthelminthic treatment. Tables S3 – S4 exclude samples taken during first captures, at which time individuals have not received anthelminthic or control treatments. Tables S5 – S6 replace anthelminthic treatment with the number of times each individual was treated before sample collection.
2. Tables S2, S4, and S6 aim to adjust for inter-plate variation in corticosterone values. Here, each sample value was divided by the corticosterone value of its inter-plate control sample and then multiplied by the plate-wide average corticosterone value. This brings all corticosterone values towards the study-wide average and minimizes inter-plate variation.

Readers should note that anthelminthic treatment never significantly predicts corticosterone values, regardless of type of anthelminthic variable / subset and regardless of adjustments for inter-plate variation in corticosterone values.

| **Table S2:** All corticosterone values, corticosterone values adjusted for inter-plate variation (n = 268 samples from 100 individuals). | | | | | |
| --- | --- | --- | --- | --- | --- |
| **Fixed Effects** | **Estimate ± SE** | **df** | **F-value** | **p-value** |  |
| Intercept | 8.55 ± 0.56 |  |  |  |  |
| Food Availability | 0.02 ± 0.23 | 1, 101.49 | 3.10 | 0.08 |  |
| Egg Burden | -0.09 ± 0.05 | 1, 238.04 | 1.44 | 0.23 |  |
| Drug Treatment | -0.11 ± 0.16 | 1, 75.90 | 0.49 | 0.48 |  |
| Julian Date^1^ | -2.96 ± 0.53 | 1, 214.15 | 30.09 | <0.0001* |  |
| Reproductive Status | 0.32 ± 0.17 | 1, 215.32 | 3.51 | 0.06 |  |
| Sex | 0.56 ± 0.16 | 1, 84.06 | 11.50 | 0.001* |  |
| Age | 0.81 ± 0.25 | 1, 212.04 | 10.06 | 0.002* |  |
| Site | 0.31 ± 0.16 | 1, 72.96 | 4.00 | 0.049* |  |
| Food x Worm Burden | 0.09 ± 0.06 | 1, 234.47 | 2.08 | 0.15 |  |
| ^1^Julian Date is scaled from 0 (end of May) to 1 (end of November) | | | | |  |
| **Table S3:** Subsetted to exclude pre-treatment (first capture) samples (n = 175 samples from 93 individuals). | | | | | |
| **Fixed Effects** | **Estimate ± SE** | **df** | **F-value** | **p-value** |  |
| Intercept | 7.95 ± 0.76 |  |  |  |  |
| Food Availability | 0.16 ± 0.29 | 1, 75.74 | 3.73 | 0.057 |  |
| Egg Burden | -0.08 ± 0.06 | 1, 157.30 | 0.75 | 0.39 |  |
| Drug Treatment | -0.12 ± 0.19 | 1, 57.53 | 0.40 | 0.53 |  |
| Julian Date^1^ | -3.34 ± 0.70 | 1, 151.38 | 22.33 | <0.0001* |  |
| Reproductive Status | 0.48 ± 0.20 | 1, 142.76 | 5.48 | 0.02* |  |
| Sex | 0.73 ± 0.20 | 1, 67.70 | 13.29 | 0.001* |  |
| Age | 0.72 ± 0.37 | 1, 148.44 | 3.68 | 0.057 |  |
| Site | 0.10 ± 0.18 | 1, 57.69 | 0.29 | 0.59 |  |
| Food x Worm Burden | 0.07 ± 0.08 | 1, 150.66 | 0.78 | 0.38 |  |
| ^1^Julian Date is scaled from 0 (end of May) to 1 (end of November) | | | | |  |

| **Table S4:** Subsetted to exclude pre-treatment (first capture) samples and corticosterone values adjusted for inter-plate variation (n = 175 samples from 93 individuals). | | | | | |
| --- | --- | --- | --- | --- | --- |
| **Fixed Effects** | **Estimate ± SE** | **df** | **F-value** | **p-value** |  |
| Intercept | 8.77 ± 0.75 |  |  |  |  |
| Food Availability | 0.20 ± 0.29 | 1, 77.38 | 3.60 | 0.06 |  |
| Egg Burden | -0.07 ± 0.06 | 1, 158.68 | 0.81 | 0.37 |  |
| Drug Treatment | -0.13 ± 0.19 | 1, 58.82 | 0.48 | 0.49 |  |
| Julian Date^1^ | -3.36 ± 0.69 | 1, 153.92 | 22.76 | <0.0001* |  |
| Reproductive Status | 0.46 ± 0.20 | 1, 145.76 | 5.06 | 0.03* |  |
| Sex | 0.72 ± 0.19 | 1, 68.81 | 13.11 | 0.001* |  |
| Age | 0.81 ± 0.37 | 1, 149.98 | 4.67 | 0.03* |  |
| Site | 0.10 ± 0.18 | 1, 57.92 | 0.27 | 0.60 |  |
| Food x Worm Burden | 0.06 ± 0.08 | 1, 153.24 | 0.44 | 0.51 |  |
| ^1^Julian Date is scaled from 0 (end of May) to 1 (end of November) | | | | |  |

| **Table S5:** All corticosterone values, replacing anthelminthic treatment with the number of treatments received prior to each sample (n = 268 samples from 100 individuals). | | | | |
| --- | --- | --- | --- | --- |
| **Fixed Effects** | **Estimate ± SE** | **df** | **F-value** | **p-value** |
| Intercept | 7.67 ± 0.58 |  |  |  |
| Food Availability | -0.04 ± 0.23 | 1, 99.82 | 2.77 | 0.09 |
| Egg Burden | -0.09 ± 0.05 | 1, 238.46 | 1.03 | 0.31 |
| Number of Treatments Received | 0.006 ± 0.06 | 1, 100.41 | 0.01 | 0.92 |
| Julian Date^1^ | -2.95 ± 0.59 | 1, 169.76 | 24.58 | <0.0001* |
| Reproductive Status | 0.33 ± 0.17 | 1, 220.45 | 3.49 | 0.059 |
| Sex | 0.58 ± 0.16 | 1, 83.16 | 12.10 | 0.001* |
| Age | 0.72 ± 0.25 | 1, 209.74 | 7.96 | 0.01* |
| Site | 0.31 ± 0.16 | 1, 72.65 | 3.83 | 0.054 |
| Food x Worm Burden | 0.11 ± 0.06 | 1, 233.64 | 3.05 | 0.08 |
| ^1^Julian Date is scaled from 0 (end of May) to 1 (end of November) | | | | |

| **Table S6:** All corticosterone values, replacing anthelminthic treatment with the number of anthelminthic treatments received prior to each sample, and corticosterone values adjusted for inter-plate variation (n = 268 samples from 100 individuals). | | | | |
| --- | --- | --- | --- | --- |
| **Fixed Effects** | **Estimate ± SE** | **df** | **F-value** | **p-value** |
| Intercept | 8.50 ± 0.58 |  |  |  |
| Food Availability | -0.04 ± 0.23 | 1, 101.18 | 2.86 | 0.09 |
| Egg Burden | -0.09 ± 0.05 | 1, 239.79 | 1.07 | 0.30 |
| Number of Treatments Received | 0.004 ± 0.06 | 1, 103.34 | 0.01 | 0.94 |
| Julian Date^1^ | -2.96 ± 0.59 | 1, 171.43 | 24.68 | <0.0001* |
| Reproductive Status | 0.31 ± 0.17 | 1, 222.10 | 3.35 | 0.07 |
| Sex | 0.57 ± 0.17 | 1, 83.80 | 11.52 | 0.001* |
| Age | 0.79 ± 0.25 | 1, 210.93 | 9.60 | 0.002* |
| Site | 0.30 ± 0.16 | 1, 73.24 | 3.74 | 0.057 |
| Food x Worm Burden | 0.10 ± 0.06 | 1, 234.67 | 2.55 | 0.11 |
| ^1^Julian Date is scaled from 0 (end of May) to 1 (end of November) | | | | |


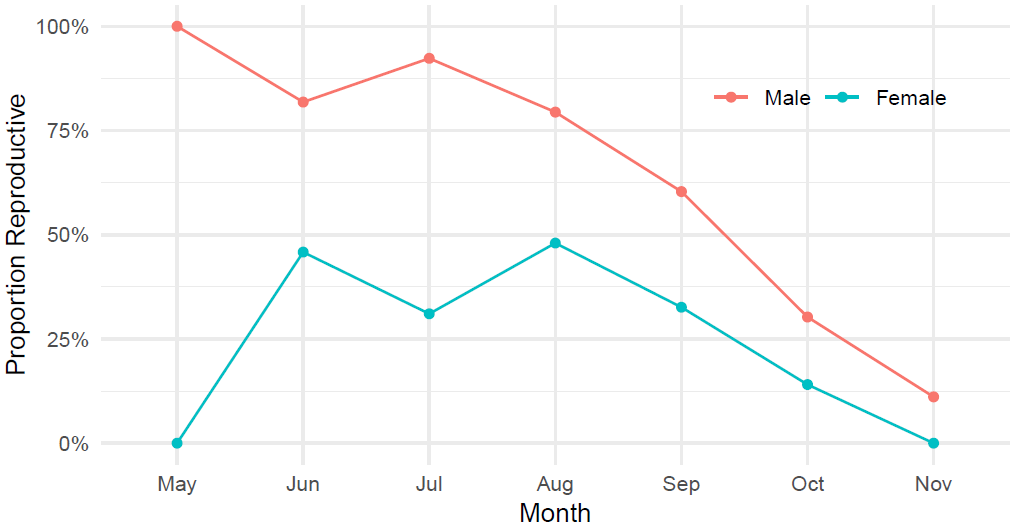


**Figure S1.** Seasonal variation in reproductive condition of wild wood mice (*Apodemus sylvaticus*) from May to November 2023. The proportion of individuals in reproductive condition is shown separately for males (red line) and females (blue line).


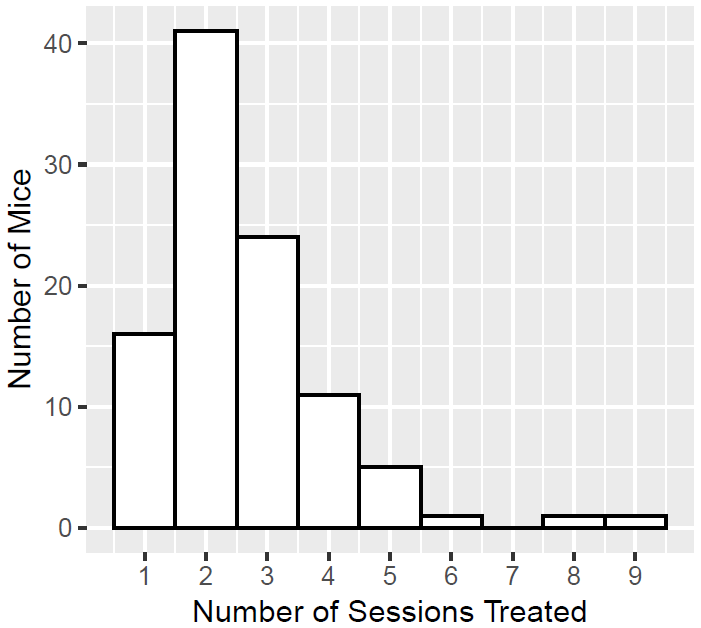


**Figure S2.** Histogram showing the number of sessions (out of 9) in which each mouse received Anthelminthic or Control treatment over the course of the study. Treatments were administered at a minimum interval of three weeks.


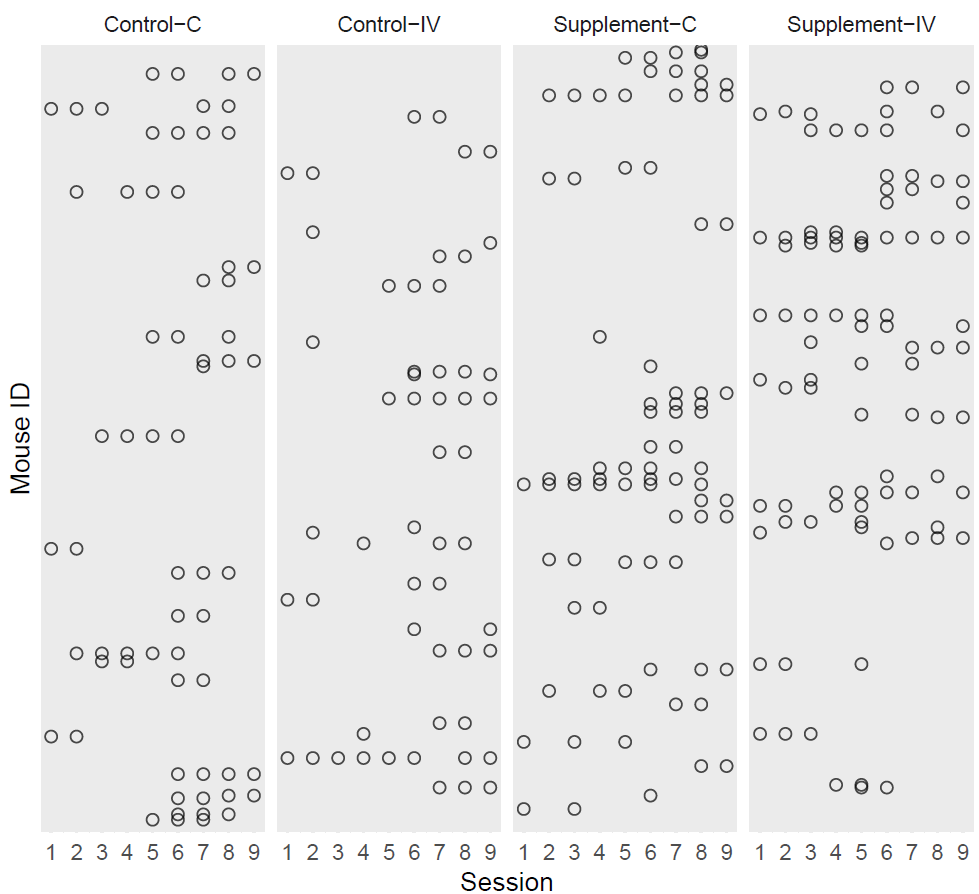


**Figure S3.** Timing of Anthelminthic or Control treatments across the 9 trapping sessions during the breeding season, separated by the combination of food supplementation and treatment group. Each row represents a unique mouse, and each dot indicates a trapping session in which the mouse received a treatment dose.

**Figures S4 – S5: Re-extraction and Dual-Assay Quantification of Faecal Corticosterone Metabolites**

To evaluate the comparability of corticosterone (CORT) measurements obtained using two different ELISA assays, we re-extracted and re-analysed a subset of 22 faecal samples collected in 2023 that were stored at –80 °C immediately after collection. Samples were assayed using: (1) the original Abcam kit (AB108821) designed to quantify unmetabolized corticosterone, and (2) the Arbor Assays kit (#K014), which is designed to measure faecal corticosterone metabolites.

Samples spanned the seasonal range and treatment structure of the original dataset. Because the initial analysis revealed a pronounced decline in corticosterone across the breeding season and a marginal increase in response to food supplementation, we selected 11 samples from early season and 11 from late season, representing a mixture of food treatment groups. No anthelminthic-treated samples were included.

All samples were extracted following the same methanol extraction protocol used for the full dataset. Briefly, faecal material was extracted overnight in methanol, after which each extract was divided into two equal aliquots and dried. One aliquot was assigned to each ELISA assay. Dried extracts were resuspended in equivalent volumes of the appropriate assay buffer for each kit. Thus, the only methodological difference between paired measurements was the assay kit employed.

As shown below (**Figures S4-S5**), corticosterone levels produced by both kits recapitulated patterns shown in the reported study-wide results. In particular, corticosterone levels were lower in later-season samples, slightly higher for mice captured on food-supplemented grids, and higher in reproductive individuals. Furthermore, the correlation between corticosterone values obtained from both kits is high (cor = 0.80, p <0.0001), suggesting that results are consistent between kits.

**Figure S4. Comparison of corticosterone levels across ELISA kits. (Left Column)** Results obtained from the original dataset reported in the main manuscript using the Abcam ELISA kit (y-axis is log(ng/g)). **(Centre Column)** Results obtained from a subset of samples run on the Arbor Assays ELISA kit. **(Right Column)** Results obtained from a subset of sample run on the original Abcam ELISA kit. We visually split these results by three variables shown to be significantly and/or marginally associated with corticosterone levels in the main text: **(First Row)** season/date, **(Second Row)** food treatment group, and **(Third Row)** reproductive status.


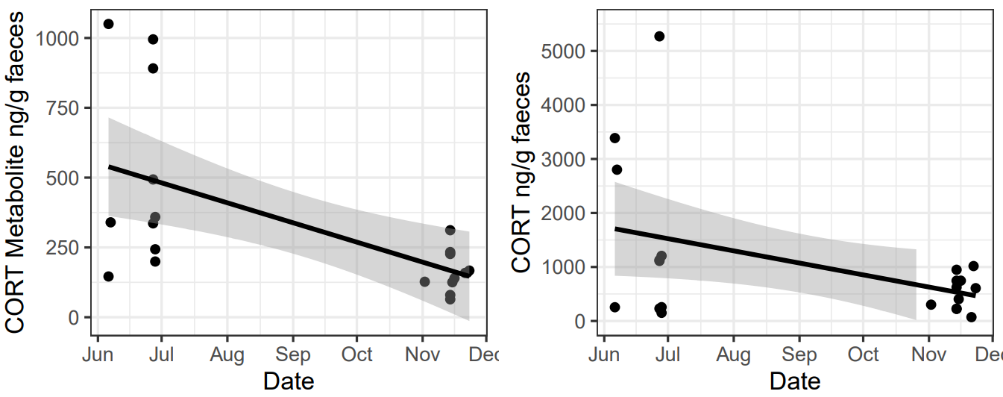

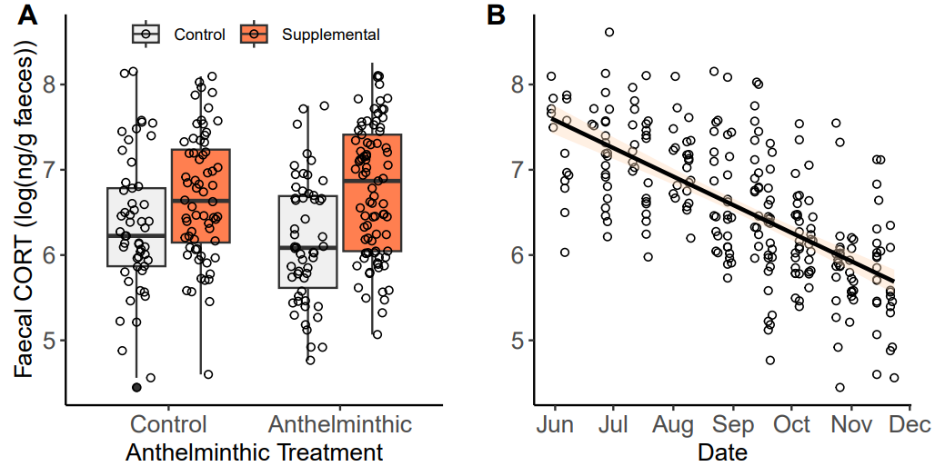


**Original Dataset**

**New Kit (Metabolites)**

**Old Kit (CORT)**


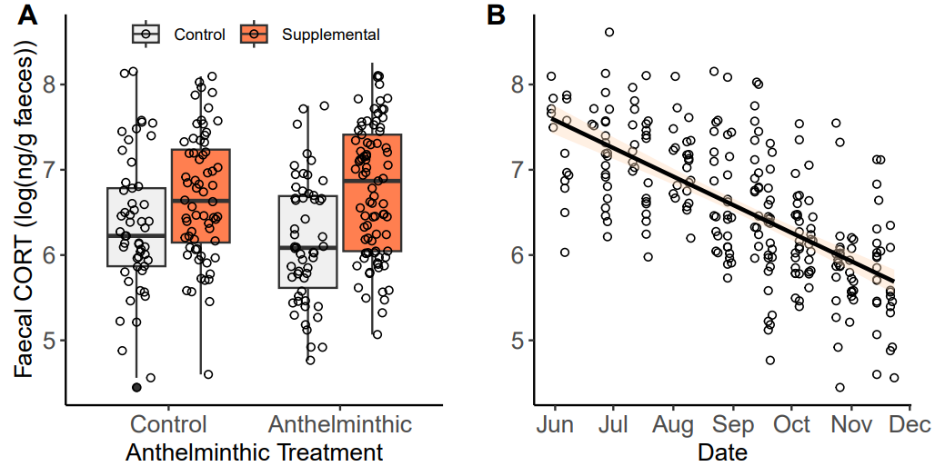

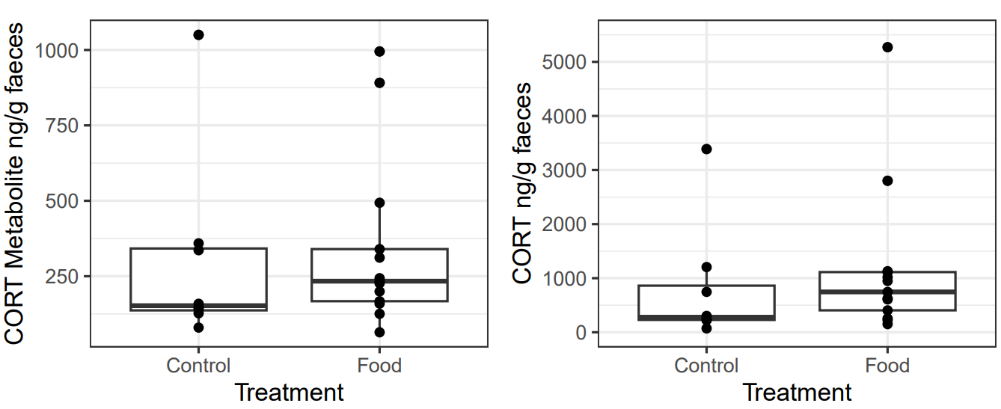


**Original Dataset**

**New Kit (Metabolites)**

**Old Kit (CORT)**


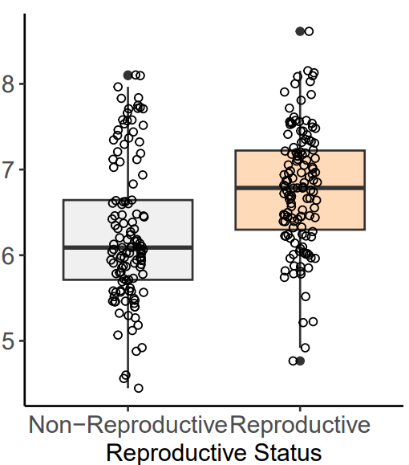


**Original Dataset**

**New Kit (Metabolites)**

**Old Kit (CORT)**


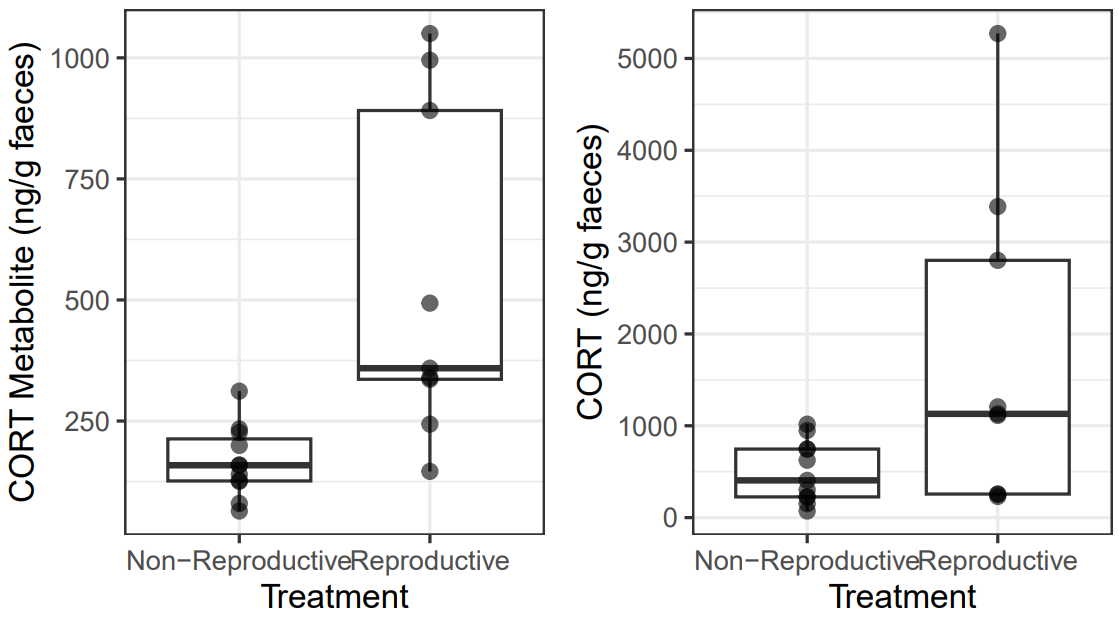


**
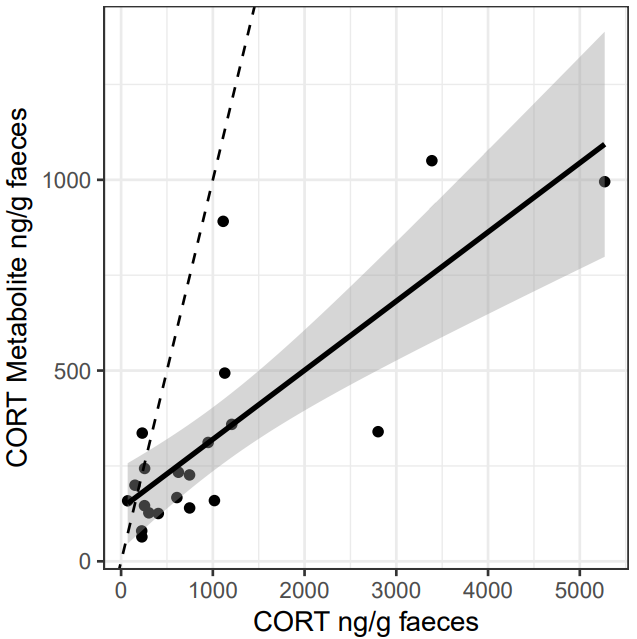
Figure S5. Correlation of faecal corticosterone metabolite values measured using the Abcam (x-axis) and Arbor Assays (y-axis) kits.** Values from the two kits are significantly correlated (cor = 0.80, p = <0.0001). The dashed line indicates a perfect 1:1 relationship between FCMs measured via each kit, and the grey shading shows the 95% confidence interval of the fitted regression.

**
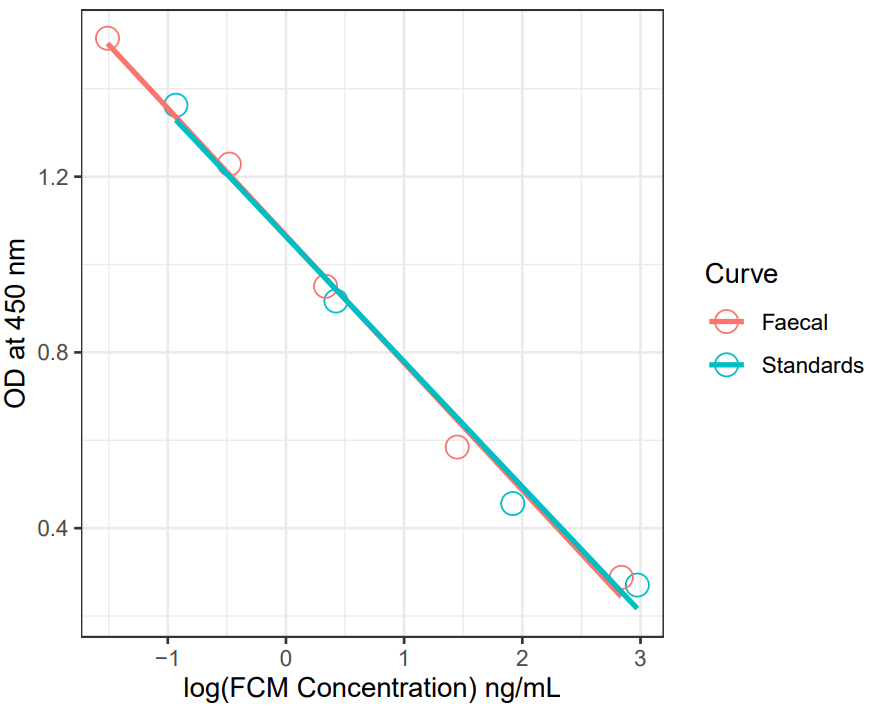
**

**Figure S6. Parallelism between faecal extracts and Abcam kit standards.**
Serial dilutions of faecal samples (“Faecal”) and kit standards (“Standards”) were used to generate response curves. Points represent observed optical density (OD) values, and lines show fitted 4-parameter logistic (4PL) curves. The curves were parallel across the assay range, indicating proportional responses to dilution. Statistical comparison of models with shared versus curve-specific slopes showed that allowing slopes to vary did not significantly improve model fit (ANOVA, *F*₁ = 5.38, *p* = 0.146), supporting the assumption of parallelism.
